# Genetic Variation in the *HSD17B1* Gene and Risk of Prostate Cancer

**DOI:** 10.1371/journal.pgen.0010068

**Published:** 2005-11-25

**Authors:** Peter Kraft, Paul Pharoah, Stephen J Chanock, Demetrius Albanes, Laurence N Kolonel, Richard B Hayes, David Altshuler, Gerald Andriole, Christine Berg, Heiner Boeing, Noel P Burtt, Bas Bueno-de-Mesquita, Eugenia E Calle, Howard Cann, Federico Canzian, Yen-Ching Chen, David E Crawford, Alison M Dunning, Heather S Feigelson, Matthew L Freedman, John M Gaziano, Ed Giovannucci, Carlos Alberto Gonzalez, Christopher A Haiman, Goran Hallmans, Brian E Henderson, Joel N Hirschhorn, David J Hunter, Rudolf Kaaks, Timothy Key, Loic Le Marchand, Jing Ma, Kim Overvad, Domenico Palli, Malcolm C Pike, Elio Riboli, Carmen Rodriguez, Wendy V Setiawan, Meir J Stampfer, Daniel O Stram, Gilles Thomas, Michael J Thun, Ruth Travis, Antonia Trichopoulou, Jarmo Virtamo, Sholom Wacholder

**Affiliations:** 1 Program in Molecular and Genetic Epidemiology, Harvard School of Public Health, Boston, Massachusetts, United States of America; 2 CRC Human Cancer Genetics Research Group, University of Cambridge, Cambridge, United Kingdom; 3 Core Genotyping Facility, National Cancer Institute, Gaithersburg, Maryland, United States; 4 Division of Cancer Epidemiology and Genetics, National Cancer Institute, Rockville, Maryland, United States of America; 5 Cancer Research Center, University of Hawaii, Honolulu, Hawaii, United States of America; 6 Broad Institute at Harvard and MIT, Cambridge, Massachusetts, United States of America; 7 Washington University, St. Louis, Missouri, United States of America; 8 Division of Cancer Prevention, National Cancer Institute, Bethesda, Maryland, United States of America; 9 Department of Epidemiology, German Institute of Human Nutrition, Potsdam, Germany; 10 Whitehead/MIT Center for Genome Research, Cambridge, Massachusetts, United States of America; 11 Centre for Nutrition and Health, National Institute for Public Health and the Environment, Bilthoven, the Netherlands; 12 Department of Epidemiology and Surveillance Research, American Cancer Society, National Home Office, Atlanta, Georgia, United States of America; 13 Fondation Jean Dausset, Centre d'Etude du Polymorphisme Humain, Paris, France; 14 Genetic Susceptibility Group, International Agency for Research on Cancer, Lyon, France; 15 Anschutz Cancer Pavillon, Aurora, Colorado, United States of America; 16 Department of Oncology, University of Cambridge, Cambridge, United Kingdom; 17 Whitehead Institute for Biomedical Research, Cambridge, Massachusetts, United States of America; 18 Department of Medicine, Division of Aging, Brigham and Women's Hospital, Boston, Massachusetts, United States of America; 19 Department of Nutrition, Harvard School of Public Health, Boston, Massachusetts, United States of America; 20 Catalan Institute of Oncology, L'Hospitalet de Llobregat, Barcelona, Spain; 21 Keck School of Medicine, University of Southern California, Los Angeles, California, United States of America; 22 Department of Public Health and Clinical Medicine, Umea University, Umea, Sweden; 23 Hormones and Cancer Group, International Agency for Research on Cancer, Lyon, France; 24 Epidemiology Unit, Cancer Research UK, Oxford, United Kingdom; 25 Channing Laboratory, Brigham and Women's Hospital, Department of Medicine, Harvard Medical School, Boston, Massachusetts, United States of America; 26 Department of Clinical Epidemiology, Aalborg Hospital, Aarhus University Hospital, Aalborg, Denmark; 27 Molecular and Nutritional Epidemiology Unit, CSPO-Scientific Institute of Tuscany, Florence, Italy; 28 Unit of Nutrition and Cancer**,** International Agency for Research on Cancer, Lyon, France; 29 American Cancer Society, Atlanta, Georgia, United States of America; 30 Department of Preventive Medicine, University of Southern California/Norris Comprehensive Cancer Center, Los Angeles, California, United States of America; 31 Department of Epidemiology, Harvard School of Public Health, Boston, Massachusetts, United States of America; 32 Division of Biostatistics and Genetic Epidemiology, Department of Preventive Medicine, Keck School of Medicine**,** University of Southern California, Los Angeles, California, United States of America; 33 Department of Hygiene and Epidemiology, University of Athens Medical School, Athens, Greece; 34 Cancer Prevention Unit, National Public Health Institute, Helsinki, Finland; University of Michigan, United States of America

## Abstract

Steroid hormones are believed to play an important role in prostate carcinogenesis, but epidemiological evidence linking prostate cancer and steroid hormone genes has been inconclusive, in part due to small sample sizes or incomplete characterization of genetic variation at the locus of interest. Here we report on the results of a comprehensive study of the association between *HSD17B1* and prostate cancer by the Breast and Prostate Cancer Cohort Consortium, a large collaborative study. *HSD17B1* encodes 17β-hydroxysteroid dehydrogenase 1, an enzyme that converts dihydroepiandrosterone to the testosterone precursor Δ5-androsterone-3β,17β-diol and converts estrone to estradiol. The Breast and Prostate Cancer Cohort Consortium researchers systematically characterized variation in *HSD17B1* by targeted resequencing and dense genotyping; selected haplotype-tagging single nucleotide polymorphisms (htSNPs) that efficiently predict common variants in U.S. and European whites, Latinos, Japanese Americans, and Native Hawaiians; and genotyped these htSNPs in 8,290 prostate cancer cases and 9,367 study-, age-, and ethnicity-matched controls. We found no evidence that *HSD17B1* htSNPs (including the nonsynonymous coding SNP S312G) or htSNP haplotypes were associated with risk of prostate cancer or tumor stage in the pooled multiethnic sample or in U.S. and European whites. Analyses stratified by age, body mass index, and family history of disease found no subgroup-specific associations between these *HSD17B1* htSNPs and prostate cancer. We found significant evidence of heterogeneity in associations between *HSD17B1* haplotypes and prostate cancer across ethnicity: one haplotype had a significant (*p* < 0.002) inverse association with risk of prostate cancer in Latinos and Japanese Americans but showed no evidence of association in African Americans, Native Hawaiians, or whites. However, the smaller numbers of Latinos and Japanese Americans in this study makes these subgroup analyses less reliable. These results suggest that the germline variants in *HSD17B1* characterized by these htSNPs do not substantially influence the risk of prostate cancer in U.S. and European whites.

## Synopsis

Steroid hormones such as estrogen and testosterone are hypothesized to play a role in the development of cancer. This is the first substantive paper from the Breast and Prostate Cancer Cohort Consortium, a large, international study designed to assess the effect of variation in genes that influence hormone production and activity on the risk of breast and prostate cancer. The investigators first constructed a detailed map of genetic variation spanning *HSD17B1,* a gene involved in the production of estrogen and testosterone. This enabled them to efficiently measure common variation across the whole gene, capturing information about both known variants with a plausible function and unknown variants with an unknown function. Because of the results with a large number of study participants, the investigators could rule out strong associations between common *HSD17B1* variants and risk of prostate cancer among U.S. and European whites. While this sheds some light on the carcinogenic effects of one enzyme involved in the complex process of steroid hormone production, it remains to be determined whether variants in other genes play a more important role or if the combined effects of several genes within these pathways have a larger impact.

## Introduction

Prostate cancer is a leading cause of mortality and morbidity in both western Europe and the United States, where it is the most commonly diagnosed nondermatological cancer and is the second leading cause of cancer death in men. Although epidemiological investigations over several decades have studied exogenous risk factors for prostate cancer, including diet, occupation, and sexually transmitted agents, the only established risk factors for this disease are age, ethnicity, and family history of prostate cancer.

A large body of evidence suggests that inherited genetic susceptibility plays an important role in prostate cancer etiology [1,2]. The risk of prostate cancer in first-degree male relatives of affected individuals is about twice that for the general population, with greater risks for those with an increased number of affected family members [3,4]. Twin studies show that the majority of this excess familial risk is due to inherited factors [5]. Unlike other cancers, however, high-penetrance prostate cancer susceptibility genes have not been consistently identified. Numerous studies have found suggestive linkage signals, but these have been difficult to replicate. Similarly, studies of candidate genes suggested by early linkage studies (e.g., *ELAC2, RNASEL,* and *MSR1*) have provided inconsistent evidence for association [1,2]. Carriers of mutations in *BRCA1* and *BRCA2* reportedly have increased risk of prostate cancer [6,7], but these mutations are rare and account for only a small fraction of excess familial risk. All of this evidence suggests multiple common variants that moderately increase prostate cancer risk have yet to be identified. Genes encoding proteins involved in hormone biosynthesis are plausible candidates for such low-penetrance variants.

Steroid hormones are believed to play an important role in prostate carcinogenesis for several reasons. First, androgens are essential for prostate maturation and functional integrity. Second, androgen ablation is standard therapy for metastatic prostate cancer. Third, androgens are generally needed to induce prostate cancer in animal models.

The results of studies of serum concentration of androgens in relation to prostate cancer risk have been somewhat inconsistent. A meta-analysis of eight prospective serum-based studies showed modest increases in prostate cancer risk associated with androstanediol glucuronide levels but not with testosterone, non–steroid hormone–binding globulin-bound testosterone, dihydrotestosterone, or androstendione levels [8], although the largest prospective study found increased risk with increasing levels of testosterone, after adjustment for serum sex hormone–binding globulin [9].

One endogenous source of variation in serum or tissue concentrations of steroid hormones may be functional variants in genes related to their synthesis and catabolism. Pursuing this line of reasoning, several investigators have examined polymorphisms in some of these genes [10–12]. For example, the missense mutation A49T in the steroid 5α-reductase type 2 gene *(SRD5A2)* increases enzyme activity for converting testosterone to the more potent dihydrotestosterone [13]. Several studies found that men who carry the *T* allele are at increased risk for prostate cancer, although these results are not conclusive [14]. Another study found that the *T* allele is associated with tumor aggressiveness [15]. Shorter repeats of the (CAG)_n_ trinucleotide in the androgen receptor gene *(AR)* are associated with increased androgen response gene transactivation and show modest increases in risk in some, but not all, studies [10,16,17]. The CYP3A4*1B allele has also been consistently associated with prostate cancer onset and severity, although the functional impact of this allele remains controversial [18]. A number of studies have found evidence that combinations of multiple polymorphisms in steroid-pathway genes increase risk of prostate cancer [11,18,19], but these studies have had low power to detect gene-gene interactions (increasing the likelihood that these results are due to chance [20]) and to the best of our knowledge have not been replicated.

Thus, the combined evidence provides several clues about the role of steroid hormones in prostate cancer yet does not permit definitive conclusions, partly because of limitations in previous epidemiological study designs. Serum hormone studies are limited because serum levels may not reflect prostate tissue levels, and the studies have generally been small (case sample sizes have varied from 16 to 222). Further, hormone interrelationships (e.g., estrogen-androgen balance) have not been considered in detail. Genetic studies have assessed only a few of the potentially important gene variants in similarly underpowered investigations; and gene-gene and gene-environment interrelationships have yet to be effectively examined.

The Breast and Prostate Cancer Cohort Consortium (BPC3), a large, multicenter collaborative study, aims to examine the role of steroidal hormones in prostate cancer by comprehensively measuring variation in more than 30 genes involved in the steroidal hormone pathway and their associated receptors in 8,301 prostate cancer cases and 9,373 controls (unpublished data). The BPC3 has adopted a multistage approach combining genomic, statistical, and epidemiological methods that involves targeted resequencing in a multiethnic sample of 190 advanced breast and prostate cancer cases, followed by genotyping a dense set of common single nucleotide polymorphisms (SNPs) across a region spanning the gene in a multiethnic sample of 349 cancer-free subjects. These genotyping data are used to assess patterns of linkage disequilibrium and select efficient haplotype-tagging SNPs (htSNPs), which are then genotyped on the cases and controls in the main study. The BPC3 provides excellent statistical power to detect modest associations between common genetic variants and risk of prostate cancer and to assess the joint effect of genetic variation and other established risk factors.

Here we report on the association between prostate cancer and the gene encoding 17β-hydroxysteroid dehydrogenase 1 (17β-HSD-1), *HSD17B1,* which is situated on chromosome 17q21 near *BRCA1.* 17β-HSD-1 plays a role in estrogen and testosterone biosynthesis. We hypothesize that germline variation in *HSD17B1* may lead to variation in 17β-HSD-1 activity. Specifically, 17β-HSD-1 catalyzes the conversion of estrone to the more reactive estradiol and may play a role in the conversion of adrenal-derived dehydroepiandrosterone to Δ5-androsterone-3β,17β-diol [21]. Δ5-Androsterone-3β,17β-diol has estrogenic activity and peroxisomal proliferation activity, via peroxisome proliferative activated receptor α [22]. It also acts as a substrate for conversion to testosterone by 3β-hydroxysteroid dehydrogenase/Δ^4^-Δ^5^ isomerase. Testosterone in turn can be metabolized to the more functionally active dihydrotestosterone by steroid-5-α-reductase [21,23]. Dehydroepiandrosterone, estrogens (estrone, estradiol), and androgens (testosterone, dihydrotestosterone) are hormones that affect prostate physiology and possibly carcinogenesis [24,25]. Thus, increased activity of 17β-HSD-1 may increase the levels of these hormones and the risk of prostate cancer. Functionally active 17β-HSD-1 is expressed in the testis [26], the primary site of testosterone synthesis. Although initial studies found evidence of *HSD17B1* expression in prostate tissue [21,27], more recent studies of prostate cancer cell lines have found small amounts of the longer of the two *HSD17B1* transcripts, which does not appear to correlate with 17β-HSD-1 protein levels [28–32].

While previous studies have evaluated whether germline variation in *HSD17B1* is associated with breast or endometrial cancer [33–36], this is the first large prospective study to assess *HSD17B1* in relation to prostate cancer among men from several ethnicities.

## Results

Table 1 shows demographic and other characteristics of cases and controls from the seven cohorts. Most study subjects were U.S. or European whites (75%), followed by African Americans (10%), Latinos (7%), Japanese Americans (5%), and Native Hawaiians (1%). Of the 8,301 prostate cancer cases and 9,373 controls sent for genotyping, at least one SNP was successfully genotyped for 8,290 (>99.8%) cases and 9,367 (>99.9%) controls, with 7,713 (93%) cases and 8,715 (93%) controls genotyped for all four markers. Among those subjects with data on both genotypes and family history, 832 (14%) cases and 555 (9%) controls reported a father or a brother with prostate cancer. Cases and controls were comparable with respect to age, body mass index (BMI [kg/m^2^]), and height. Stage information was available on 71% of genotyped prostate cancer cases, and among these, 1,312 (22%) had advanced disease (defined as stage C or D disease at diagnosis or death due to prostate cancer). Gleason score was recorded for 66% of genotyped cases, with 990 cases (18% of those with Gleason scores exhibiting scores of eight or greater).

**Table 1:**
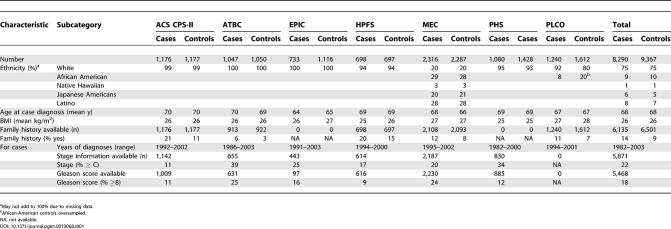
Characteristics of the Study Population by Study, BPC3

**Figure 1:**
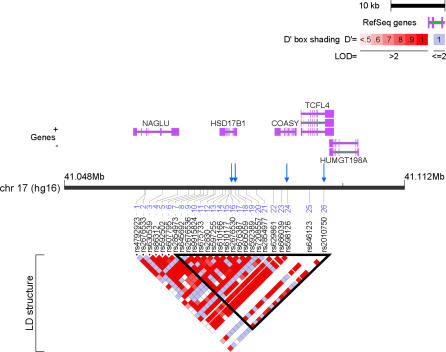
A Scale Map of the 26 SNPs Genotyped in the MEC Screening Panel and a Plot of the Pattern of Linkage Disequilibrium among Them (in Whites) The four tag SNPs are markers with arrows, and the block of high linkage disequilibrium and limited haplotype diversity spanning *HSD17B1* is highlighted.

Resequencing exons in 190 advanced cancer cases identified two novel nonsynonymous coding SNPs, one of which was seen more than once. (For more detailed resequencing results, see Gene_Summary_Table.xls available under “Genes: Data and Haplotypes” at http://www.uscnorris.com/Core/DocManager/OpenFile.aspx?DocID=9394.) The latter SNP and 25 common SNPs spanning a 42-kb region including *HSD17B1* were genotyped in a multiethnic reference panel of 349 cancer-free subjects. Nineteen of these 26 SNPs formed a block of high linkage disequilibrium (Figure 1) that spans *HSD17B1*, including (5′ to 3′) the pseudogene *HSD17BP1,* the promoter region, and the gene *TCFL4.* We found three common haplotypes (>5% frequency) within this block among whites in the reference panel, with a cumulative frequency above 83% (Table S1). We chose four SNPs that predict these common haplotypes in whites with a minimum *R_h_* ^2^ of 82% (Figure 1 and Table 2); we required that the nonsynonymous coding SNP S312G (rs605059) be included in the set of htSNPs. These four htSNPs also predicted common haplotypes (>5% frequency) in African Americans, Native Hawaiians, Japanese Americans, and Latinos with a minimum *R_h_* ^2^ above 80% (Table S1). However, among African Americans, the cumulative frequency of common haplotypes was only 62%. To achieve a cumulative frequency above 70% in African Americans (i.e., to predict an additional two haplotypes with an *R_h_* ^2^ >80%), additional htSNPs were needed. Because we did not genotype these extra SNPs for this analysis, our current analyses of African Americans are principally informative for haplotypes with frequency above 5%. None of the SNP genotype frequencies showed evidence of deviation from Hardy-Weinberg equilibrium at the 0.001 level among controls in any of the cohorts (stratified by ethnicity).

**Table 2:**
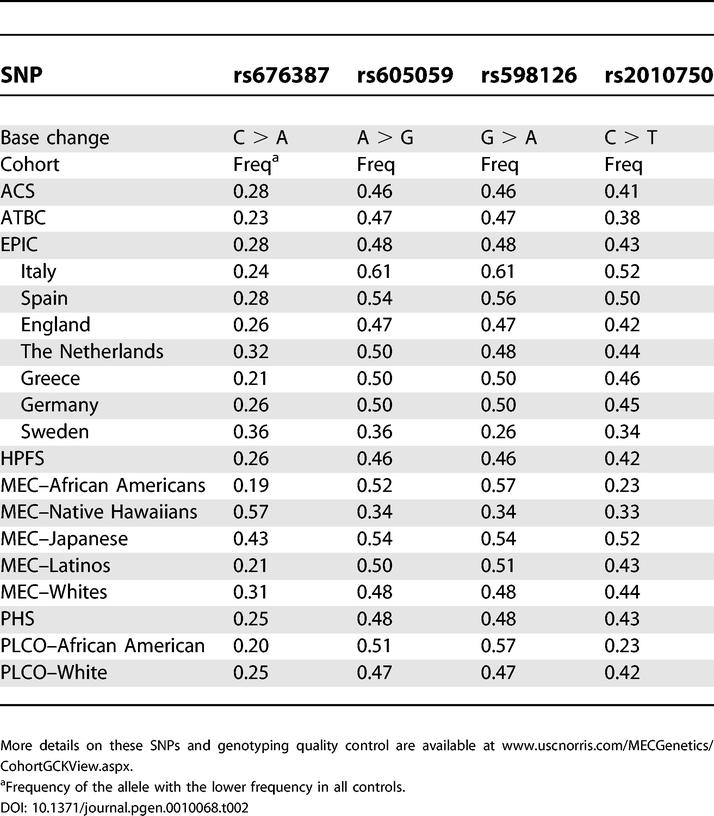
Characteristics of the htSNPs for *HSD17B1*

Four htSNP haplotypes had frequencies above 5% in white controls, with a cumulative frequency above 99% (Table 3). Haplotype frequencies were similar for whites across cohorts (Table S2), while some differences in haplotype frequencies were seen among whites, African Americans, Japanese Americans, and Native Hawaiians. Consistent with the greater genetic diversity in African Americans, we found one haplotype (CAAC) that was common only in African Americans (Table 3).

**Table 3:**
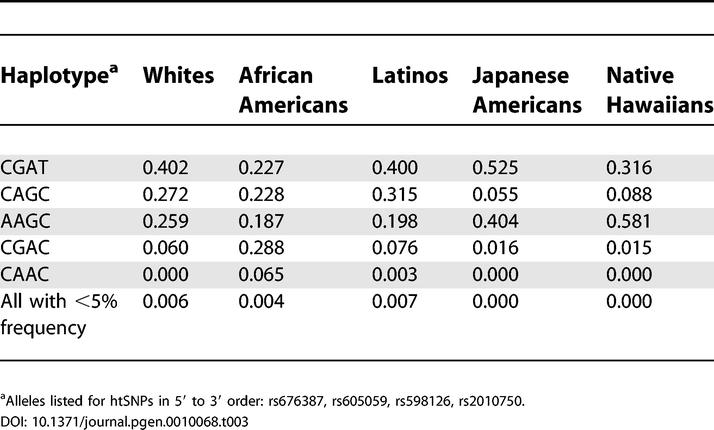
Haplotype Frequencies in Controls, by Ethnicity

**Figure 2:**
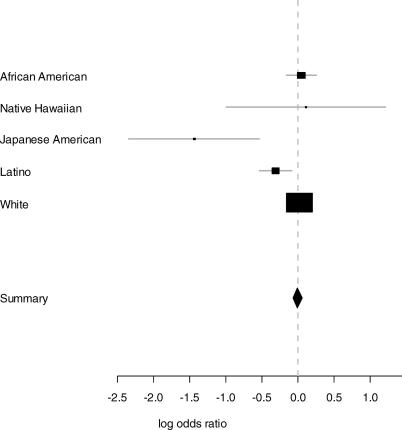
Plot of Log Odds Ratios for CAGC (Relative to All Other Haplotypes) under an Additive Model The boxes are proportional to the inverse of the parameter estimate variance; larger boxes denote more precise estimates. The error bars mark 99% confidence intervals.

Global tests of association between *HSD17B1* haplotypes and prostate cancer were not significant (likelihood-ratio test [LRT] χ^2^ = 5.25, 5 d.f., *p* = 0.39 for analysis using all subjects; LRT χ^2^ = 6.45, 5 d.f., *p* = 0.22 for analysis restricted to whites; see Table 4). However, the test for heterogeneity in haplotype odds ratios across ethnicities was significant (LRT χ^2^ = 44.66, 15 d.f., *p* < 0.0001). While no haplotype showed evidence of association with prostate cancer risk in African Americans, whites, or Native Hawaiians, haplotype CAGC was significantly associated with decreased prostate cancer risk in Latinos and Japanese Americans (Figure 2; more detailed cohort- and ethnicity-specific results are given in Table S3). The test for heterogeneity across cohorts in haplotype odds ratios among whites was not significant (LRT χ^2^ = 37.82, 23 d.f., *p* = 0.03).

**Table 4:**
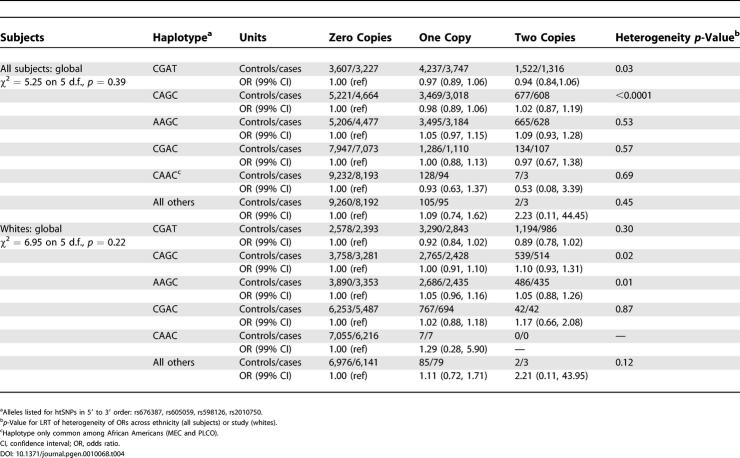
HSD17B1 Haplotypes and Prostate Cancer Risk, BPC3

Genotype-specific odds ratios for the four SNPs tested are shown in Table 5 for analyses restricted to whites and for analyses pooling all subjects. There was no evidence of an association between the nonsynonymous S312G SNP and prostate cancer (*p* = 0.40 for analysis using all subjects and *p* = 0.09 for analysis restricted to whites). None of the other SNPs showed any evidence of association with prostate cancer at the 0.01 level, and none of the SNP odds ratios showed significant evidence of heterogeneity across ethnicity (Table S4).

**Table 5:**
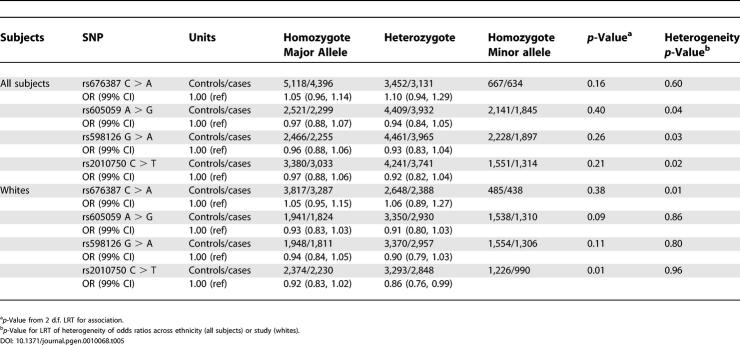
HSD17B1 htSNPs and Prostate Cancer Risk, BPC3

We calculated stratum-specific SNP and haplotype odds ratios for strata defined by family history (at least one first-degree relative diagnosed with prostate cancer versus none), age at time of diagnosis or time of diagnosis of the matched case for controls (≤65 versus >65 years old), and BMI (<25, ≥25 but <30, >30). None of these stratum-specific tests of association were significant at the 0.01 level, and tests for departures from multiplicative interaction model (tests for “statistical interaction”) were also nonsignificant (Tables S5 and S6).

We also found no association between *HSD17B1* haplotypes and advanced prostate cancer (Table 6).

**Table 6:**
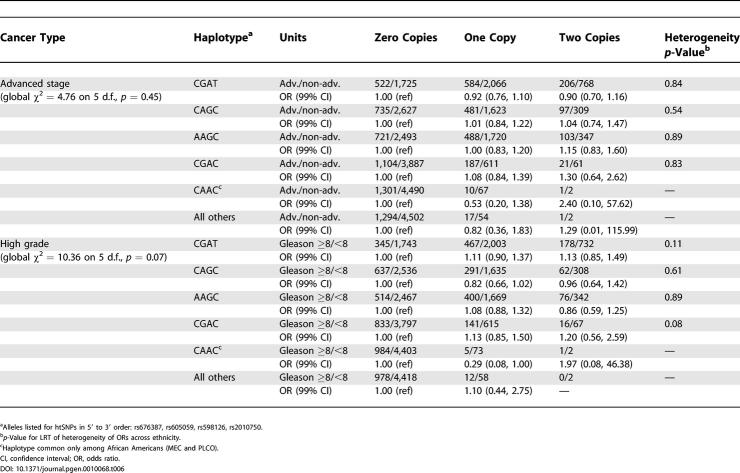
*HSD17B1* Haplotypes and Risk of Advanced Stage (≥C) and High-Grade (Gleason ≥8) Prostate Cancer among All Cases

## Discussion

After comprehensively screening *HSD17B1* for variation in U.S. and European whites, we found no evidence of association between prostate cancer and common variants in *HSD17B1.* We observed that haplotype odds ratios for association with prostate cancer differed across ethnicity, with the CAGC haplotype showing a significant (*p* < 0.01) inverse association with prostate cancer effects in Latinos and Japanese Americans. However, the smaller sample size in these subgroups limits our power to detect an effect of the observed magnitude, leading to an increase in the probability that these results are false positives. We found no evidence that the odds ratios associated with common haplotypes or SNPs differed by cohort (among whites), family history, age, or BMI. We also found no evidence that common variants in *HSD17B1* were related to disease severity among cases.

A major advantage of the BPC3 haplotype-tagging approach is that it allows a cost-effective approach to the identification of common susceptibility alleles across the entire gene region. This includes putative functional variants such as nonsynonymous coding SNPs as well as variants of unknown function in intronic and 5′ and 3′ untranslated regions. In the case of *HSD17B1,* there is evidence that several upstream regions participate in the regulation of *HSD17B1* expression [28]. All of these lie well within the region of high linkage disequilibrium spanning *HSD17B1;* hence, common variants in these regulatory regions are accurately predicted by the four htSNPs analyzed here.

Another major strength of the BPC3 is its unprecedented sample size. With 8,290 cases and 9,367 controls, there is greater than 90% power to detect a dominant or log-additive odds ratio of 1.3 for an allele with 5% frequency at the 0.001 level, even after accounting for loss of effective sample size due to the haplotype-tagging approach. The large sample size of the BPC3 allows adequately powered investigation of differences in genetic effect by established or hypothesized prostate cancer risk factors. For example, there is still greater than 90% power to detect a stratum-specific dominant odds ratio of 1.7 for a 5% frequency variant at the 0.001 level when the stratum consists of only 20% of the total sample.

A limitation of the study is the inability of the assayed htSNPs to adequately capture several haplotypes in African Americans that have a frequency just below 5%, so the cumulative frequency of the haplotypes that are effectively predicted in this group by the htSNPs is only 60%. Moreover, power to detect possible associations between prostate cancer and genetic variation in *HSD17B1* unique to nonwhite ethnicities is limited in this study, given the smaller sample sizes available for these groups. For example, power to detect the observed log-additive effect sizes for haplotype CAGC in Japanese Americans at the 0.01 level is approximately 59% (68% for Latinos). Thus, assuming prior probabilities of causality for this *HSD17B1* haplotype of 1%, the false-positive probabilities for these associations are 64% for Japanese Americans and 59% for Latinos; assuming a more conservative prior probability of 0.1%, the false-positive probabilities are 95% for Japanese Americans and 94% for Latinos. Further analysis to assess whether *HSD17B1* is associated with risk for prostate cancer in nonwhite ethnic groups will require larger samples accumulated through longer follow-up or new cohorts.

Another potential limitation of this study is that results are reported for a single gene. Prostate cancer risk may be a complex function of genotypes across several genes involved in steroid hormone metabolism [37]. For example, a mutation in one gene may only increase risk in the presence of a mutation in a different gene. Although a gene involved in such gene-gene interactions can be discovered using a marginal test (ignoring the other genes) [38], incorporating information about other genes may improve power to detect association [39]. As the BPC3 will eventually measure variation in over 30 genes related to the steroid hormone pathway, it will have the opportunity to investigate the combined contribution of multiple genes to the risk of prostate cancer.

In the present study, the absence of an association between *HSD17B1* haplotypes and prostate cancer suggests that we can rule out large or moderate associations between common *HSD17B1* variants and risk of prostate cancer among U.S. and European whites. If any variants affect the risk of prostate cancer, they are likely to have small effects or low frequency and are unlikely to contribute significantly to the overall incidence of prostate cancer in these populations. While this sheds some light on the clinical effects of one enzyme involved in the complex process of steroid synthesis and catabolism, it remains to be determined whether variants in other genes in steroidal hormone pathways play a more important role or if the combined effects of several genes within these pathways have a larger impact. The BPC3 plans to investigate these questions by comprehensively measuring variation in more than 30 genes involved in the steroidal hormone pathway and their associated receptors. Last, this study underscores the importance of large, cooperative consortia in evaluating the contribution of germline genetic variation to a common cancer, such as prostate cancer.

## Materials and Methods

### Study population.

The BPC3 has been described in detail elsewhere (unpublished data). For prostate cancer, the study combines the resources of seven large cohort studies of men: the American Cancer Society Cancer Prevention Study II (ACS CPS-II) [40], the Alpha-Tocopherol, Beta-Carotene Cancer Prevention (ATBC) Study [41], the EPIC Cohort (itself comprising cohorts from Denmark, Great Britain, Germany, Greece, Italy, the Netherlands, Spain, and Sweden) [42], the Health Professionals Follow-up Study (HPFS) [43], the Hawaii/Los Angeles Multi-ethnic Cohort Study (MEC) [44], the Physicians Health Study (PHS) [45], and the Prostate, Lung, Colorectal, and Ovarian (PLCO) Cancer Screening Trial [46]. With the exception of the MEC, these cohorts are composed predominantly of whites of European descent. We do not have information on ethnicity or ancestry beyond country of residence for EPIC and have classified all EPIC participants as white. We anticipate that the number of EPIC participants of non-European ancestry is small. We plan further population genetic studies will to verify this, using the large number of SNPs the BPC3 will have genotyped. The MEC contributes African American, Latino, Japanese Americans, and Native Hawaiian cases and controls recruited from Los Angeles and Hawaii. The PLCO also includes over 650 African American subjects. We distinguish Spanish EPIC participants from MEC Latino participants, because the latter are principally of Mexican and Central American origin, with origins in European, Native American, and African populations [47,48].

Cases of prostate cancer were identified through population-based cancer registries or self-report confirmed by medical records. The BPC3 data for prostate cancer consist of a series of matched nested case-control studies from each cohort; controls were matched to cases on a number of potentially confounding factors, including age, ethnicity, and region of recruitment. For the current investigation, prostate cancer cases were matched to available controls by age in 5-year intervals, ethnicity, and cohort.

### SNP discovery and htSNP selection.

We used a multistage approach to characterize genetic variation in and around *HSD17B1* in our large sample of cases and controls. First, *HSD17B1* exons in 190 advanced breast and prostate cancer cases were resequenced at the Broad Institute to discover novel coding SNPs. Then we genotyped a set of SNPs across a 42-kb region spanning *HSD17B1* in a multiethnic reference sample to determine patterns of linkage disequilibrium and select htSNPs. This set consisted of 25 common (allele frequency >5%) SNPs selected from public databases and one nonsynonymous SNP discovered during resequencing; these 26 SNPs covered the target region at a density of one SNP per 1.6 kb. The target region included the gene *N*-acetylglucosaminidase alpha *(NAGLU)* and the pseudogene for *HSD17B1 (HSD17BP1),* both 5′ of *HSD17B1,* and coenzyme A synthase *(COASY)* and transcription factor-like 4 *(TCFL4),* both 3′ of *HSD17B1* (see Figure 1). The reference sample consisted of equal numbers (70 each) of whites, African Americans, Latinos, and Japanese Americans and 69 Native Hawaiians.

We identified a region of high linkage disequilibrium and low haplotype diversity spanning *HSD17B1* using the algorithm of Gabriel et al. [49] as implemented in Haploview [50]. Haplotype-tagging SNPs were then chosen in these regions based on *R_h_* ^2^, a measure of the correlation between observed haplotypes and those predicted on the basis of htSNP genotypes [51]. This approach is based on the observation that within blocks of high linkage disequilibrium and limited haplotype diversity, common SNPs are highly correlated with common haplotypes [49]. Finally, we genotyped these htSNPs in BPC3 cases and controls and tested for association between htSNP haplotypes and disease.

### Genotyping.

The 26 SNPs were genotyped in the multiethnic reference panel at the Broad Institute using Sequenom and Illumina platforms. Genotyping of cases and controls was performed in 4 laboratories using a fluorescent 5′ endonuclease assay and ABI PRISM 7900 for sequence detection (TaqMan; Applied Biosystems, Foster City, California, United States). Based on sequence information, TaqMan assays were designed for each SNP and synthesized in four separate batches of 12,000 reactions for the roughly 48,000 needed to complete the study of *HSD17B1* in BPC3 breast and prostate cancer samples. Initial quality control was performed at the manufacturer (Applied Biosystems); an additional 500 test reactions were run by the Cohort Consortium on the multiethnic reference panel; greater than 99.5% concordance was observed across genotyping platforms. (Assay characteristics for the four htSNPs for *HSD17B1* are available on the public Website: http://www.uscnorris.com/mecgenetics/CohortGCKView.aspx.) Sequence validation for each SNP assay was performed and 100% concordance observed (http://snp500cancer.nci.nih.gov) [52]. To assess interlaboratory variation, each center ran assays on a designated set of 94 samples from the SNP 500 cancer panel, showing completion and concordance rates of greater than 99% [52]. The internal quality of genotype data at each center was assessed by 5% to 10% blinded samples in duplicate or triplicate (depending on study).

### Statistical analysis.

For each SNP, we used conditional logistic regression to simultaneously estimate the odds ratio for disease associated with carrying one copy of the minor allele relative to carrying no copies and the odds ratio associated with carrying two copies relative to carrying no copies. We estimated haplotype-specific odds ratios using an expectation-substitution approach to account for haplotype uncertainty given unphased genotype data [53,54]. Haplotype frequencies and subject-specific expected haplotype indicators were calculated separately for each cohort (and country or ethnicity within cohort). To test the global null hypothesis of no association between variation in *HSD17B1* haplotypes and risk of prostate cancer, we used an LRT comparing a model with additive effects on the log odds scale for each common haplotype (treating the most common haplotype as the referent) to the intercept-only model. We considered haplotypes with greater than 5% frequency in at least one cohort or ethnic group to be “common.” All other haplotypes were pooled into a separate “rare haplotypes” category.

Although the matched analysis accounts for heterogeneity in risk-factor prevalence across study, we also tested for heterogeneity in odds ratio estimates across studies that might result from slightly different matching criteria or case definitions using an LRT. We also tested for heterogeneity in odds ratio estimates across ethnicity using an LRT that compares the model with common additive effects for each haplotype (except the referent) to the model with distinct additive effects for each ethnicity where the expected numbers of the haplotype in cases and controls under the null were above five. Thus, the three common haplotypes among Native Hawaiians contributed two ethnicity-specific haplotype effects; the five common haplotypes and the pooled rare haplotypes among African Americans contribute five. To assess whether other risk factors for prostate cancer modify the association with haplotype, we calculated risk stratum–specific odds ratios and tested for departures from a multiplicative interaction model. We performed case-only analyses to test for association between *HSD17B1* variants and advanced prostate cancer (as defined above).

We calculated 99% confidence intervals and test for significance associations at the 0.01 level to minimize the chance of both false-positive and false-negative results. An upper bound on the probability of a false positive was estimated roughly as α(1 – π)/[α(1 – π) + π(1 – β)], where π is the prior probability that a variant has a relative risk of *R* or greater, α is the test size, and 1 – β is the power [20]. Our study has greater than 99% power to detect a dominant or log-additive odds ratio of 1.3 for an allele with 5% frequency at the 0.001 level. Thus, when α = 0.01, the probability of a false positive is 8% for the very optimistic prior probability of a 10% chance that *HSD17B1* is associated with prostate cancer. The false-negative report probability, defined as β π/[(1 – α)(1 – π) + π β], is only 0.1% in this situation. For a prior probability of 1 in 100, the false-positive and false-negative report probabilities are 50% and 0.01%, respectively. Thus, for a range of priors, the probability that we would fail to reject at the .01 level if *HSD17B1* were truly associated with disease is small. Power for individual SNPs and haplotypes was calculated using Quanto (http://hydra.usc.edu/gxe/), assuming an effective sample size of *N  R_h_* ^2^ to adjust for the loss in power inherent in genotyping surrogate tagging SNPs. Here *N* is the nominal sample size and *R_h_* ^2^ is the design threshold of 0.8; this is somewhat conservative, as the achieved *R_h_* ^2^ can be well above the threshold.

## Supporting Information

Table S1 Haplotype Frequencies and htSNP Performance in MEC Reference Sample (61 KB DOC)

Table S2 htSNP Haplotype Frequencies by Cohort Among Whites and African Americans. (106 KB DOC)

Table S3 Tests of Haplotype-Prostate Cancer Association and Haplotype Odds Ratios, by Study (Whites) and Ethnicity (439 KB DOC)

Table S4 Tests of Association between Individual htSNPs and Prostate Cancer and Odds Ratio Estimates, by Study (Whites) and Ethnicity (175 KB DOC)

Table S5 Tests of Haplotype-Prostate Cancer Association and Haplotype Odds Ratios, Stratified by Age, Family History of Prostate Cancer, and BMI (346 KB DOC)

Table S6 Tests of Between-Individual htSNPs and Prostate Cancer and Odds Ratio Estimates, Stratified by Age, Family History of Prostate Cancer, and BMI (285 KB DOC)

### Accession Numbers

The OMIM (http://www.ncbi.nlm.nih.gov/OMIM) accession numbers for genes mentioned in this paper are *AR* (313700), *BRCA1* (113705), *BRCA2* (600185), CYP3A4*1B (124010), *ELAC2* (605367), *HSD17B1* (109684), *RNASEL* (180435), *MSR1* (153622), *NAGLU* (252920), *SRD5A2* (607306), and *TCFL4* (602976). The HGNC (http://www.gene.ucl.ac.uk/cgi-bin/nomenclature/searchgenes.pl) accession number for *COASY* is 29932.

## Abbreviations

BMI: body mass index
BPC3: Breast and Prostate Cancer Cohort Consortium
17β-HSD-1: 17β-hydroxysteroid dehydrogenase 1
htSNP: haplotype-tagging single nucleotide polymorphism
LRT: likelihood-ratio test
SNP: single nucleotide polymorphism

## Note Added in Proof

The Breast and Prostate Cancer Cohort Consortium (BPC3) study, cited in this paper as unpublished data, is now in press [55]
