# Supplementary material for: Genetic Variation in the HSD17B1 Gene and Risk of Prostate Cancer
Source: PLoS Genet. 2005 Nov 25;1(5):e68. doi: 10.1371/journal.pgen.0010068 (PMC1287955; doi:10.1371/journal.pgen.0010068)
Supplement: Supplementary file 1 [file pgen.0010068.st001.doc]

Table S1. Haplotype frequencies and htSNP performance in MEC reference sample

a. Whites

| Haplotype | Frequency | Cumulative Frequency | *R_h_*^2^ |
| --- | --- | --- | --- |
| ** * * |  |  |  |
| 4331144334431244243 | 0.37 | 0.37 | 0.97 |
| 2311324433241233421 | 0.27 | 0.64 | 0.81 |
| 2331144313431244223 | 0.20 | 0.84 | 1.00 |
| 2213322433241433423 | 0.04 | 0.88 | 0.46 |
| 2213324433241433423 | 0.02 | 0.90 | 0.27 |

b. African-Americans

| Haplotype | Frequency | Cumulative Frequency | *R_h_*^2^ |
| --- | --- | --- | --- |
| ** * * |  |  |  |
| 2311324433241233421 | 0.25 | 0.25 | 0.75 |
| 2331144313431244223 | 0.18 | 0.43 | 0.73 |
| 4331144334431244243 | 0.12 | 0.55 | 0.89 |
| 2331144333431243423 | 0.07 | 0.62 | 0.96 |
| 2331144333231243423 | 0.05 | 0.67 | 0.16 |
| 2331142333241243423 | 0.05 | 0.72 | 0.16 |
| 2211124433231233423 | 0.03 | 0.75 | 0.11 |
| 2311322433241233421 | 0.03 | 0.78 | 0.08 |

c. Native Hawaiians

| Haplotype | Frequency | Cumulative Frequency | *R_h_*^2^ |
| --- | --- | --- | --- |
| ** * * |  |  |  |
| 4331144334431244243 | 0.49 | 0.49 | 0.98 |
| 2311324433241233421 | 0.26 | 0.74 | 0.74 |
| 2331144313431244223 | 0.08 | 0.82 | 0.88 |
| 2311324433441233421 | 0.05 | 0.87 | 0.93 |
| 2311322433241233421 | 0.04 | 0.92 | 0.10 |
| 2213324433241433423 | 0.02 | 0.94 | 0.59 |

Notes: Haplotypes (above 2% frequency) given as strings of alleles for the block containing HSD17B1, i.e. contiguous SNPs rs4792923 through rs2010750 (see Figure 1 for full list of intervening SNPs). Alleles coded as "1" for "A", "2" for "C", "3" for "G" and "4" for "T". htSNPs are marked by asterisks.

Table S1 (continued). Haplotype frequencies and htSNP performance in MEC reference sample

d. Japanese

| Haplotype | Frequency | Cumulative Frequency | *R_h_*^2^ |
| --- | --- | --- | --- |
| ** * * |  |  |  |
| 4331144334431244243 | 0.53 | 0.53 | 0.97 |
| 2311324433241233421 | 0.32 | 0.85 | 0.70 |
| 2311322433241233421 | 0.04 | 0.89 | 0.06 |
| 2311324433441233421 | 0.04 | 0.93 | 1.00 |
| 2311122433241233421 | 0.02 | 0.95 | 0.04 |

e. Latinos

| Haplotype | Frequency | Cumulative Frequency | *R_h_*^2^ |
| --- | --- | --- | --- |
| ** * * |  |  |  |
| 2311324433241233421 | 0.30 | 0.30 | 0.65 |
| 2331144313431244223 | 0.27 | 0.57 | 0.88 |
| 4331144334431244243 | 0.21 | 0.78 | 0.94 |
| 2311322433241233421 | 0.10 | 0.88 | 0.17 |
| 2311324433441233421 | 0.03 | 0.91 | 0.97 |
