# Supplementary material for: Genetic Variation in the HSD17B1 Gene and Risk of Prostate Cancer
Source: PLoS Genet. 2005 Nov 25;1(5):e68. doi: 10.1371/journal.pgen.0010068 (PMC1287955; doi:10.1371/journal.pgen.0010068)
Supplement: Supplementary file 2 [file pgen.0010068.st002.doc]

Haplotype frequencies in whites - ACS

| **Haplotype** | **Controls (Observed)** | **Controls (Frequency)** | **Cases (Observed)** | **Cases (Frequency)** |
| --- | --- | --- | --- | --- |
| h2314 | 948 | 0.407 | 936 | 0.402 |
| h2132 | 613 | 0.263 | 660 | 0.283 |
| h2112 | 1 | 0.000 | 1 | 0.000 |
| h2312 | 123 | 0.053 | 121 | 0.052 |
| h1132 | 635 | 0.273 | 606 | 0.260 |
|  | 10 | 0.004 | 7 | 0.003 |

Haplotype frequencies in whites - ATBC

| **Haplotype** | **Controls (Observed)** | **Controls (Frequency)** | **Cases (Observed)** | **Cases (Frequency)** |
| --- | --- | --- | --- | --- |
| h2314 | 789 | 0.376 | 761 | 0.364 |
| h2132 | 646 | 0.308 | 593 | 0.283 |
| h2112 | 0 | 0.000 | 0 | 0.000 |
| h2312 | 182 | 0.087 | 185 | 0.088 |
| h1132 | 474 | 0.226 | 543 | 0.259 |
|  | 8 | 0.004 | 11 | 0.005 |

Haplotype frequencies in whites - EPIC

| **Haplotype** | **Controls (Observed)** | **Controls (Frequency)** | **Cases (Observed)** | **Cases (Frequency)** |
| --- | --- | --- | --- | --- |
| h2314 | 953 | 0.427 | 587 | 0.400 |
| h2132 | 534 | 0.239 | 383 | 0.262 |
| h2112 | 1 | 0.001 | 2 | 0.002 |
| h2312 | 107 | 0.048 | 79 | 0.054 |
| h1132 | 611 | 0.274 | 403 | 0.275 |
|  | 26 | 0.012 | 11 | 0.007 |

Haplotype frequencies in whites - HPFS

| **Haplotype** | **Controls (Observed)** | **Controls (Frequency)** | **Cases (Observed)** | **Cases (Frequency)** |
| --- | --- | --- | --- | --- |
| h2314 | 508 | 0.389 | 514 | 0.391 |
| h2132 | 378 | 0.289 | 367 | 0.280 |
| h2112 | 1 | 0.001 | 0 | 0.000 |
| h2312 | 65 | 0.050 | 75 | 0.057 |
| h1132 | 340 | 0.260 | 336 | 0.255 |
|  | 13 | 0.010 | 22 | 0.017 |

Haplotype frequencies in whites - MEC

| **Haplotype** | **Controls (Observed)** | **Controls (Frequency)** | **Cases (Observed)** | **Cases (Frequency)** |
| --- | --- | --- | --- | --- |
| h2314 | 399 | 0.441 | 389 | 0.426 |
| h2132 | 186 | 0.205 | 238 | 0.261 |
| h2112 | 0 | 0.000 | 2 | 0.002 |
| h2312 | 38 | 0.043 | 50 | 0.055 |
| h1132 | 279 | 0.309 | 234 | 0.257 |
|  | 2 | 0.002 | 0 | 0.000 |

Haplotype frequencies in whites - PHS

| **Haplotype** | **Controls (Observed)** | **Controls (Frequency)** | **Cases (Observed)** | **Cases (Frequency)** |
| --- | --- | --- | --- | --- |
| h2314 | 1018 | 0.382 | 757 | 0.370 |
| h2132 | 772 | 0.289 | 581 | 0.284 |
| h2112 | 1 | 0.000 | 2 | 0.001 |
| h2312 | 193 | 0.072 | 139 | 0.068 |
| h1132 | 663 | 0.249 | 540 | 0.264 |
|  | 20 | 0.007 | 28 | 0.014 |

Haplotype frequencies in whites - PLCO

| **Haplotype** | **Controls (Observed)** | **Controls (Frequency)** | **Cases (Observed)** | **Cases (Frequency)** |
| --- | --- | --- | --- | --- |
| h2314 | 1062 | 0.411 | 872 | 0.382 |
| h2132 | 715 | 0.276 | 632 | 0.277 |
| h2112 | 2 | 0.001 | 0 | 0.000 |
| h2312 | 144 | 0.056 | 129 | 0.057 |
| h1132 | 655 | 0.253 | 641 | 0.281 |
|  | 8 | 0.003 | 5 | 0.002 |

Haplotype frequencies in African Americans - MEC

| **Haplotype** | **Controls (Observed)** | **Controls (Frequency)** | **Cases (Observed)** | **Cases (Frequency)** |
| --- | --- | --- | --- | --- |
| h2314 | 297 | 0.229 | 325 | 0.239 |
| h2132 | 298 | 0.230 | 313 | 0.230 |
| h2112 | 82 | 0.063 | 72 | 0.053 |
| h2312 | 383 | 0.295 | 367 | 0.270 |
| h1132 | 234 | 0.180 | 281 | 0.207 |
|  | 3 | 0.003 | 2 | 0.002 |

Haplotype frequencies in African Americans - PLCO

| **Haplotype** | **Controls (Observed)** | **Controls (Frequency)** | **Cases (Observed)** | **Cases (Frequency)** |
| --- | --- | --- | --- | --- |
| h2314 | 143 | 0.224 | 43 | 0.216 |
| h2132 | 144 | 0.225 | 53 | 0.267 |
| h2112 | 43 | 0.068 | 11 | 0.056 |
| h2312 | 176 | 0.276 | 53 | 0.263 |
| h1132 | 128 | 0.200 | 39 | 0.197 |
|  | 5 | 0.007 | 0 | 0.002 |
