# Supplementary material for: Genetic Variation in the HSD17B1 Gene and Risk of Prostate Cancer
Source: PLoS Genet. 2005 Nov 25;1(5):e68. doi: 10.1371/journal.pgen.0010068 (PMC1287955; doi:10.1371/journal.pgen.0010068)
Supplement: Supplementary file 3 [file pgen.0010068.st003.doc]

AFRICAN AMERICANS - GLOBAL CHI-SQUARE 5.15 ON 5 D.F. P=0.40

Print Data Set WORK.OUTTABLEBLA1 Haplotype	Chi-Square	DF	P-value	Count	Case	Control	Odds Ratio Estimate	Lower 99% Confidence Limit for Odds Ratio	Upper 99% Confidence Limit for Odds Ratio	
h2314	0.12	2	0.9435	0	462	586	1.00	.	.	
	.	.	.	1	267	324	1.03	0.77	1.36	
	.	.	.	2	50	58	1.06	0.61	1.84	
h2132	0.43	2	0.8059	0	464	584	1.00	.	.	
	.	.	.	1	266	326	1.03	0.78	1.36	
	.	.	.	2	50	58	1.15	0.66	1.99	
h2112	1.42	2	0.4918	0	700	849	1.00	.	.	
	.	.	.	1	77	112	0.88	0.57	1.35	
	.	.	.	2	3	7	0.53	0.08	3.37	
h2312	1.60	2	0.4488	0	422	493	1.00	.	.	
	.	.	.	1	297	392	0.89	0.67	1.17	
	.	.	.	2	61	83	0.84	0.52	1.38	
h1132	2.50	2	0.2863	0	503	646	1.00	.	.	
	.	.	.	1	233	282	1.03	0.77	1.37	
	.	.	.	2	44	40	1.46	0.79	2.73	
blk1nhap5	4.88	2	0.0870	0	777	960	1.00	.	.	
	.	.	.	1	3	8	0.39	0.05	3.30	
	.	.	.	2	0	0	I	0.00	I	


NATIVE HAWAIIANS - GLOBAL CHI-SQUARE 0.25 ON 2 D.F. P=0.88

Print Data Set WORK.OUTTABLEHAW1 Haplotype	Chi-Square	DF	P-value	Count	Case	Control	Odds Ratio Estimate	Lower 99% Confidence Limit for Odds Ratio	Upper 99% Confidence Limit for Odds Ratio	
h2314	0.29	2	0.8658	0	31	30	1.00	.	.	
	.	.	.	1	33	33	0.97	0.39	2.44	
	.	.	.	2	7	5	1.36	0.26	6.96	
h2132	0.07	1	0.7892	0	57	56	1.00	.	.	
	.	.	.	1	14	12	1.12	0.37	3.42	
	.	.	.	2	0	0	.	.	.	
h1132	0.56	2	0.7546	0	15	11	1.00	.	.	
	.	.	.	1	34	35	0.71	0.22	2.33	
	.	.	.	2	22	22	0.75	0.21	2.68	


JAPANESE AMERICANS - GLOBAL CHI-SQUARE 25.31 ON 3 D.F. P<0.0001

Print Data Set WORK.OUTTABLEJAP1 Haplotype	Chi-Square	DF	P-value	Count	Case	Control	Odds Ratio Estimate	Lower 99% Confidence Limit for Odds Ratio	Upper 99% Confidence Limit for Odds Ratio	
h2314	0.40	2	0.8192	0	107	112	1.00	.	.	
	.	.	.	1	229	222	1.08	0.70	1.67	
	.	.	.	2	126	136	0.99	0.61	1.60	
h2132	24.47	2	0.0000	0	454	427	1.00	.	.	
	.	.	.	1	7	35	0.20	0.07	0.60	
	.	.	.	2	1	8	0.12	0.01	1.94	
h2312	1.57	2	0.4560	0	449	455	1.00	.	.	
	.	.	.	1	13	15	0.88	0.32	2.41	
	.	.	.	2	0	0	0.00	0.00	I	
h1132	4.24	2	0.1199	0	138	170	1.00	.	.	
	.	.	.	1	228	220	1.29	0.87	1.90	
	.	.	.	2	96	80	1.43	0.87	2.37	


LATINOS - GLOBAL CHI-SQUARE 12.26 ON 5 D.F. P=0.03

Print Data Set WORK.OUTTABLELAT1 Haplotype	Chi-Square	DF	P-value	Count	Case	Control	Odds Ratio Estimate	Lower 99% Confidence Limit for Odds Ratio	Upper 99% Confidence Limit for Odds Ratio	
h2314	6.67	2	0.0356	0	193	237	1.00	.	.	
	.	.	.	1	324	303	1.32	0.95	1.84	
	.	.	.	2	130	108	1.45	0.95	2.22	
h2132	13.06	2	0.0015	0	354	306	1.00	.	.	
	.	.	.	1	255	275	0.81	0.60	1.10	
	.	.	.	2	37	67	0.46	0.26	0.83	
h2112	1.12	2	0.5703	0	641	644	1.00	.	.	
	.	.	.	1	5	4	1.25	0.19	8.06	
	.	.	.	2	0	0	.	.	.	
h2312	0.40	2	0.8169	0	558	557	1.00	.	.	
	.	.	.	1	84	84	1.05	0.68	1.64	
	.	.	.	2	4	7	0.71	0.14	3.68	
h1132	0.57	2	0.7523	0	400	414	1.00	.	.	
	.	.	.	1	219	211	1.04	0.76	1.42	
	.	.	.	2	27	23	1.24	0.57	2.69	
blk1nhap5	0.97	2	0.6165	0	636	638	1.00	.	.	
	.	.	.	1	10	10	1.17	0.33	4.13	
	.	.	.	2	0	0	I	0.00	I	


ACS - GLOBAL CHI-SQUARE 3.28 ON 4 D.F. P=0.51

Print Data Set WORK.OUTTABLEACS1 Haplotype	Chi-Square	DF	P-value	Count	Case	Control	Odds Ratio Estimate	Lower 99% Confidence Limit for Odds Ratio	Upper 99% Confidence Limit for Odds Ratio	
h2314	5.13	2	0.0770	0	432	400	1.00	.	.	
	.	.	.	1	529	582	0.83	0.65	1.05	
	.	.	.	2	203	183	1.01	0.74	1.39	
h2132	3.21	2	0.2005	0	591	622	1.00	.	.	
	.	.	.	1	488	472	1.08	0.86	1.35	
	.	.	.	2	86	70	1.35	0.86	2.10	
h2312	0.17	2	0.9189	0	1047	1046	1.00	.	.	
	.	.	.	1	116	116	0.99	0.69	1.43	
	.	.	.	2	2	3	0.69	0.07	7.21	
h1132	0.85	2	0.6542	0	640	620	1.00	.	.	
	.	.	.	1	443	454	0.94	0.75	1.18	
	.	.	.	2	81	90	0.88	0.58	1.35	
blk1nhap5	0.42	2	0.8098	0	1158	1155	1.00	.	.	
	.	.	.	1	7	10	0.73	0.20	2.60	
	.	.	.	2	-0	-0	.	.	.	


ATBC - GLOBAL CHI-SQUARE 8.05 ON 4 D.F. P=0.09

Print Data Set WORK.OUTTABLEATB1 Haplotype	Chi-Square	DF	P-value	Count	Case	Control	Odds Ratio Estimate	Lower 99% Confidence Limit for Odds Ratio	Upper 99% Confidence Limit for Odds Ratio	
h2314	6.61	2	0.0368	0	406	416	1.00	.	.	
	.	.	.	1	521	479	1.12	0.88	1.43	
	.	.	.	2	120	155	0.79	0.55	1.14	
h2132	3.78	2	0.1509	0	545	502	1.00	.	.	
	.	.	.	1	411	449	0.84	0.66	1.07	
	.	.	.	2	91	99	0.86	0.57	1.29	
h2312	0.11	2	0.9456	0	869	874	1.00	.	.	
	.	.	.	1	171	170	1.01	0.74	1.37	
	.	.	.	2	7	6	1.20	0.29	5.05	
h1132	7.17	2	0.0277	0	563	625	1.00	.	.	
	.	.	.	1	424	376	1.26	0.99	1.60	
	.	.	.	2	60	49	1.33	0.78	2.26	
blk1nhap5	0.54	2	0.7618	0	1036	1042	1.00	.	.	
	.	.	.	1	11	8	1.41	0.42	4.69	
	.	.	.	2	-0	-0	.	.	.	


EPIC - GLOBAL CHI-SQUARE 6.09 ON 4 D.F. P=0.19

Print Data Set WORK.OUTTABLEEPI1 Haplotype	Chi-Square	DF	P-value	Count	Case	Control	Odds Ratio Estimate	Lower 99% Confidence Limit for Odds Ratio	Upper 99% Confidence Limit for Odds Ratio	
h2314	2.67	2	0.2635	0	271	372	1.00	.	.	
	.	.	.	1	338	534	0.87	0.66	1.15	
	.	.	.	2	125	210	0.82	0.57	1.17	
h2132	2.67	2	0.2625	0	403	646	1.00	.	.	
	.	.	.	1	277	406	1.09	0.84	1.42	
	.	.	.	2	53	64	1.36	0.82	2.26	
h2312	2.55	2	0.2795	0	660	1012	1.00	.	.	
	.	.	.	1	67	101	1.03	0.67	1.59	
	.	.	.	2	6	3	2.97	0.47	18.64	
h1132	0.25	2	0.8844	0	385	593	1.00	.	.	
	.	.	.	1	294	435	1.04	0.80	1.35	
	.	.	.	2	55	88	0.96	0.60	1.55	
blk1nhap5	2.72	2	0.2563	0	722	1090	1.00	.	.	
	.	.	.	1	11	26	0.57	0.21	1.56	
	.	.	.	2	0	0	165E84	0.00	I	


HPFS - GLOBAL CHI-SQUARE 2.63 ON 4 D.F. P=0.62

Print Data Set WORK.OUTTABLEHPF1 Haplotype	Chi-Square	DF	P-value	Count	Case	Control	Odds Ratio Estimate	Lower 99% Confidence Limit for Odds Ratio	Upper 99% Confidence Limit for Odds Ratio	
h2314	2.62	2	0.2695	0	256	242	1.00	.	.	
	.	.	.	1	288	314	0.86	0.63	1.18	
	.	.	.	2	113	97	1.08	0.70	1.65	
h2132	3.19	2	0.2030	0	351	327	1.00	.	.	
	.	.	.	1	245	274	0.83	0.61	1.13	
	.	.	.	2	61	52	1.10	0.64	1.87	
h2312	1.20	2	0.5478	0	588	591	1.00	.	.	
	.	.	.	1	63	59	1.09	0.66	1.79	
	.	.	.	2	6	3	2.07	0.30	14.27	
h1132	0.39	2	0.8219	0	365	354	1.00	.	.	
	.	.	.	1	248	259	0.94	0.70	1.28	
	.	.	.	2	44	41	1.06	0.58	1.94	
blk1nhap5	3.99	2	0.1359	0	637	640	1.00	.	.	
	.	.	.	1	18	13	1.17	0.40	3.41	
	.	.	.	2	2	0	776.21	0.00	1978E9	


MEC - GLOBAL CHI-SQUARE 11.47 ON 3 D.F. P=0.01

Print Data Set WORK.OUTTABLEMEC1 Haplotype	Chi-Square	DF	P-value	Count	Case	Control	Odds Ratio Estimate	Lower 99% Confidence Limit for Odds Ratio	Upper 99% Confidence Limit for Odds Ratio	
h2314	1.48	2	0.4772	0	155	136	1.00	.	.	
	.	.	.	1	216	233	0.83	0.56	1.24	
	.	.	.	2	87	83	0.94	0.57	1.56	
h2132	7.94	2	0.0188	0	253	291	1.00	.	.	
	.	.	.	1	170	137	1.45	0.99	2.12	
	.	.	.	2	34	24	1.60	0.77	3.33	
h2312	2.23	2	0.3278	0	412	415	1.00	.	.	
	.	.	.	1	40	36	1.11	0.59	2.10	
	.	.	.	2	5	1	4.05	0.23	71.22	
h1132	6.57	2	0.0375	0	251	213	1.00	.	.	
	.	.	.	1	177	198	0.76	0.53	1.10	
	.	.	.	2	29	41	0.57	0.29	1.14	


PHS - GLOBAL CHI SQUARE 6.87 ON 4 D.F. P=0.14

Print Data Set WORK.OUTTABLEPHS1 Haplotype	Chi-Square	DF	P-value	Count	Case	Control	Odds Ratio Estimate	Lower 99% Confidence Limit for Odds Ratio	Upper 99% Confidence Limit for Odds Ratio	
h2314	1.82	2	0.4018	0	429	551	1.00	.	.	
	.	.	.	1	433	546	1.01	0.80	1.28	
	.	.	.	2	162	236	0.87	0.63	1.18	
h2132	2.14	2	0.3422	0	532	699	1.00	.	.	
	.	.	.	1	402	496	1.07	0.85	1.35	
	.	.	.	2	90	138	0.86	0.59	1.26	
h2312	0.58	2	0.7481	0	898	1162	1.00	.	.	
	.	.	.	1	114	149	0.99	0.70	1.39	
	.	.	.	2	13	22	0.76	0.30	1.93	
h1132	1.98	2	0.3719	0	561	768	1.00	.	.	
	.	.	.	1	385	467	1.13	0.90	1.43	
	.	.	.	2	78	98	1.09	0.72	1.67	
blk1nhap5	5.75	2	0.0563	0	996	1314	1.00	.	.	
	.	.	.	1	27	19	2.18	0.93	5.15	
	.	.	.	2	1	1	0.55	0.00	411.66	


PLCO - GLOBAL CHI-SQUARE 6.15 ON 4 D.F. P=0.19

Print Data Set WORK.OUTTABLEPLC1 Haplotype	Chi-Square	DF	P-value	Count	Case	Control	Odds Ratio Estimate	Lower 99% Confidence Limit for Odds Ratio	Upper 99% Confidence Limit for Odds Ratio	
h2314	3.79	2	0.1507	0	445	461	1.00	.	.	
	.	.	.	1	519	603	0.89	0.71	1.12	
	.	.	.	2	177	230	0.80	0.59	1.09	
h2132	2.99	2	0.2237	0	606	670	1.00	.	.	
	.	.	.	1	436	531	0.91	0.73	1.14	
	.	.	.	2	98	92	1.19	0.79	1.78	
h2312	0.08	2	0.9586	0	1014	1153	1.00	.	.	
	.	.	.	1	123	136	1.02	0.73	1.44	
	.	.	.	2	3	4	0.84	0.12	6.05	
h1132	4.75	2	0.0929	0	588	716	1.00	.	.	
	.	.	.	1	463	498	1.13	0.91	1.41	
	.	.	.	2	89	79	1.37	0.90	2.10	
blk1nhap5	1.33	2	0.5137	0	1135	1286	1.00	.	.	
	.	.	.	1	5	6	0.91	0.19	4.35	
	.	.	.	2	-0	1	0.00	0.00	32E297
