# Supplementary material for: Genetic Variation in the HSD17B1 Gene and Risk of Prostate Cancer
Source: PLoS Genet. 2005 Nov 25;1(5):e68. doi: 10.1371/journal.pgen.0010068 (PMC1287955; doi:10.1371/journal.pgen.0010068)
Supplement: Supplementary file 4 [file pgen.0010068.st004.doc]

African Americans	

Print Data Set WORK.TEMP SNP	Chi-Square	DF	P-value	Genotype	Control	Case	Odds Ratio Estimate	Lower 99% Confidence Limit for Odds Ratio	Upper 99% Confidence Limit for Odds Ratio	
rs676387(A)	2.68	2	0.2617	0	640	496	1.00	.	.	
	.	.	.	1	280	228	1.02	0.76	1.36	
	.	.	.	2	39	44	1.48	0.80	2.75	
rs605059(G)	0.78	2	0.6757	0	231	192	1.00	.	.	
	.	.	.	1	430	354	0.97	0.70	1.35	
	.	.	.	2	275	208	0.89	0.62	1.28	
rs598126(A)	2.58	2	0.2757	0	179	163	1.00	.	.	
	.	.	.	1	446	355	0.82	0.58	1.17	
	.	.	.	2	336	252	0.80	0.55	1.17	
rs2010750(T)	0.12	2	0.9413	0	581	451	1.00	.	.	
	.	.	.	1	323	263	1.03	0.78	1.37	
	.	.	.	2	58	49	1.05	0.60	1.81	

Native Hawaiians	

Print Data Set WORK.TEMP SNP	Chi-Square	DF	P-value	Genotype	Control	Case	Odds Ratio Estimate	Lower 99% Confidence Limit for Odds Ratio	Upper 99% Confidence Limit for Odds Ratio	
rs676387(A)	0.63	2	0.7298	0	11	15	1.00	.	.	
	.	.	.	1	35	33	0.70	0.21	2.28	
	.	.	.	2	22	23	0.78	0.22	2.77	
rs605059(G)	0.29	2	0.8659	0	28	29	1.00	.	.	
	.	.	.	1	33	32	0.95	0.37	2.43	
	.	.	.	2	6	8	1.30	0.28	6.06	
rs598126(A)	0.26	2	0.8765	0	29	29	1.00	.	.	
	.	.	.	1	33	33	1.00	0.40	2.53	
	.	.	.	2	6	8	1.34	0.29	6.20	
rs2010750(T)	0.31	2	0.8562	0	30	30	1.00	.	.	
	.	.	.	1	33	33	1.01	0.40	2.54	
	.	.	.	2	5	7	1.41	0.27	7.22	


Japanese Americans

Print Data Set WORK.TEMP SNP	Chi-Square	DF	P-value	Genotype	Control	Case	Odds Ratio Estimate	Lower 99% Confidence Limit for Odds Ratio	Upper 99% Confidence Limit for Odds Ratio	
rs676387(A)	3.97	2	0.1373	0	170	138	1.00	.	.	
	.	.	.	1	218	226	1.28	0.87	1.90	
	.	.	.	2	80	94	1.41	0.85	2.34	
rs605059(G)	0.58	2	0.7479	0	102	96	1.00	.	.	
	.	.	.	1	216	227	1.11	0.71	1.73	
	.	.	.	2	141	131	1.00	0.61	1.63	
rs598126(A)	0.61	2	0.7377	0	101	99	1.00	.	.	
	.	.	.	1	221	230	1.07	0.69	1.68	
	.	.	.	2	141	128	0.95	0.58	1.56	
rs2010750(T)	0.42	2	0.8124	0	112	105	1.00	.	.	
	.	.	.	1	216	224	1.10	0.71	1.71	
	.	.	.	2	133	125	1.02	0.63	1.66	

Latinos

Print Data Set WORK.TEMP SNP	Chi-Square	DF	P-value	Genotype	Control	Case	Odds Ratio Estimate	Lower 99% Confidence Limit for Odds Ratio	Upper 99% Confidence Limit for Odds Ratio	
rs676387(A)	0.34	2	0.8418	0	405	393	1.00	.	.	
	.	.	.	1	210	222	1.06	0.77	1.45	
	.	.	.	2	26	29	1.12	0.54	2.32	
rs605059(G)	8.90	2	0.0117	0	179	134	1.00	.	.	
	.	.	.	1	312	334	1.45	1.01	2.09	
	.	.	.	2	146	164	1.54	1.01	2.36	
rs598126(A)	7.21	2	0.0272	0	173	133	1.00	.	.	
	.	.	.	1	318	330	1.36	0.94	1.96	
	.	.	.	2	154	176	1.52	1.00	2.31	
rs2010750(T)	6.63	2	0.0363	0	233	184	1.00	.	.	
	.	.	.	1	303	319	1.33	0.95	1.86	
	.	.	.	2	106	124	1.44	0.94	2.23	


Whites

Print Data Set WORK.TEMP SNP	Chi-Square	DF	P-value	Genotype	Control	Case	Odds Ratio Estimate	Lower 99% Confidence Limit for Odds Ratio	Upper 99% Confidence Limit for Odds Ratio	
rs676387(A)	1.92	2	0.3837	0	3817	3287	1.00	.	.	
	.	.	.	1	2648	2388	1.05	0.95	1.15	
	.	.	.	2	485	438	1.06	0.89	1.27	
rs605059(G)	4.82	2	0.0899	0	1941	1824	1.00	.	.	
	.	.	.	1	3350	2930	0.93	0.83	1.03	
	.	.	.	2	1538	1310	0.91	0.80	1.03	
rs598126(A)	4.48	2	0.1066	0	1948	1811	1.00	.	.	
	.	.	.	1	3370	2957	0.94	0.84	1.05	
	.	.	.	2	1554	1306	0.90	0.79	1.03	
rs2010750(T)	8.68	2	0.0131	0	2374	2230	1.00	.	.	
	.	.	.	1	3293	2848	0.92	0.83	1.02	
	.	.	.	2	1226	990	0.86	0.76	0.99
