# Supplementary material for: Genetic Variation in the HSD17B1 Gene and Risk of Prostate Cancer
Source: PLoS Genet. 2005 Nov 25;1(5):e68. doi: 10.1371/journal.pgen.0010068 (PMC1287955; doi:10.1371/journal.pgen.0010068)
Supplement: Supplementary file 5 [file pgen.0010068.st005.doc]

AGE LT 65 - GLOBAL CHI-SQUARE 1.06 ON 5 D.F. P = 0.96


Print Data Set WORK.TEMP1 Haplotype	Chi-Square	DF	P-value	Count	Control	Case	Odds Ratio Estimate	Lower 95% Confidence Limit for Odds Ratio	Upper 95% Confidence Limit for Odds Ratio	
h2314	0.41	2	0.8131	0	1132	828	1.00	.	.	
	.	.	.	1	1290	1015	1.03	0.91	1.17	
	.	.	.	2	437	349	1.05	0.88	1.24	
h2132	0.08	2	0.9594	0	1624	1220	1.00	.	.	
	.	.	.	1	1043	823	1.02	0.90	1.15	
	.	.	.	2	192	149	1.01	0.80	1.27	
h2112	0.20	2	0.9031	0	2800	2162	1.00	.	.	
	.	.	.	1	58	29	0.93	0.57	1.53	
	.	.	.	2	1	1	1.62	0.10	26.05	
h2312	0.44	2	0.8037	0	2388	1872	1.00	.	.	
	.	.	.	1	423	290	0.94	0.79	1.12	
	.	.	.	2	48	30	0.97	0.60	1.59	
h1132	0.23	2	0.8933	0	1561	1204	1.00	.	.	
	.	.	.	1	1087	833	0.98	0.87	1.10	
	.	.	.	2	211	155	0.96	0.76	1.21	
blk1nhap5	3.33	2	0.1894	0	2821	2165	1.00	.	.	
	.	.	.	1	37	27	1.00	0.58	1.71	
	.	.	.	2	1	0	0.00	0.00	563E34	


AGE GE 65 - GLOBAL CHI-SQUARE 9.29 ON 5 D.F. P = 0.10


Print Data Set WORK.TEMP2 Haplotype	Chi-Square	DF	P-value	Count	Control	Case	Odds Ratio Estimate	Lower 95% Confidence Limit for Odds Ratio	Upper 95% Confidence Limit for Odds Ratio	
h2314	4.24	2	0.1199	0	2468	2391	1.00	.	.	
	.	.	.	1	2941	2718	0.94	0.87	1.02	
	.	.	.	2	1085	961	0.90	0.81	1.00	
h2132	1.50	2	0.4723	0	3591	3428	1.00	.	.	
	.	.	.	1	2420	2184	0.96	0.89	1.04	
	.	.	.	2	482	458	1.02	0.89	1.18	
h2112	1.58	2	0.4539	0	6418	6004	1.00	.	.	
	.	.	.	1	70	64	0.93	0.64	1.35	
	.	.	.	2	6	2	0.38	0.07	2.01	
h2312	0.19	2	0.9076	0	5546	5175	1.00	.	.	
	.	.	.	1	862	818	1.02	0.91	1.14	
	.	.	.	2	86	77	0.96	0.69	1.33	
h1132	6.67	2	0.0356	0	3640	3259	1.00	.	.	
	.	.	.	1	2401	2338	1.09	1.01	1.17	
	.	.	.	2	453	474	1.15	1.00	1.32	
blk1nhap5	3.11	2	0.2108	0	6426	6000	1.00	.	.	
	.	.	.	1	68	68	1.12	0.78	1.61	
	.	.	.	2	1	3	11.84	0.30	469.24	


FAMILY HISTORY NEGATIVE - GLOBAL CHI-SQUARE 2.88 ON 5 D.F. P = 0.72


Print Data Set WORK.TEMP1 Haplotype	Chi-Square	DF	P-value	Count	Control	Case	Odds Ratio Estimate	Lower 99% Confidence Limit for Odds Ratio	Upper 99% Confidence Limit for Odds Ratio	
h2314	1.68	2	0.4316	0	2280	2060	1.00	.	.	
	.	.	.	1	2721	2387	0.95	0.85	1.06	
	.	.	.	2	945	856	0.97	0.83	1.12	
h2132	1.28	2	0.5276	0	3346	3037	1.00	.	.	
	.	.	.	1	2192	1893	0.96	0.86	1.07	
	.	.	.	2	409	373	1.02	0.83	1.25	
h2112	1.92	2	0.3827	0	5839	5226	1.00	.	.	
	.	.	.	1	100	75	0.95	0.61	1.47	
	.	.	.	2	7	2	0.35	0.04	2.99	
h2312	0.24	2	0.8856	0	4991	4483	1.00	.	.	
	.	.	.	1	872	748	1.00	0.86	1.17	
	.	.	.	2	83	72	1.09	0.69	1.72	
h1132	3.36	2	0.1867	0	3282	2871	1.00	.	.	
	.	.	.	1	2256	2016	1.01	0.91	1.12	
	.	.	.	2	408	416	1.15	0.94	1.40	
blk1nhap5	0.41	2	0.8129	0	5898	5257	1.00	.	.	
	.	.	.	1	47	45	1.11	0.62	1.98	
	.	.	.	2	1	2	1.80	0.06	57.35	


FAMILY HISTORY POSITIVE - GLOBAL CHI-SQUARE 0.57 ON 5 D.F. P = 0.99


Print Data Set WORK.TEMP2 Haplotype	Chi-Square	DF	P-value	Count	Control	Case	Odds Ratio Estimate	Lower 99% Confidence Limit for Odds Ratio	Upper 99% Confidence Limit for Odds Ratio	
h2314	1.20	2	0.5482	0	224	302	1.00	.	.	
	.	.	.	1	252	414	1.13	0.81	1.56	
	.	.	.	2	79	116	0.97	0.62	1.54	
h2132	1.22	2	0.5435	0	293	454	1.00	.	.	
	.	.	.	1	221	311	0.88	0.64	1.21	
	.	.	.	2	41	66	1.03	0.57	1.83	
h2112	1.27	2	0.5294	0	547	827	1.00	.	.	
	.	.	.	1	8	4	0.69	0.12	4.00	
	.	.	.	2	0	1	131423	0.00	I	
h2312	1.40	2	0.4955	0	470	712	1.00	.	.	
	.	.	.	1	71	109	1.19	0.74	1.91	
	.	.	.	2	14	11	0.77	0.24	2.44	
h1132	1.50	2	0.4734	0	311	453	1.00	.	.	
	.	.	.	1	205	330	1.12	0.81	1.53	
	.	.	.	2	39	49	0.86	0.46	1.60	
blk1nhap5	2.65	2	0.2652	0	547	822	1.00	.	.	
	.	.	.	1	8	9	0.57	0.15	2.15	
	.	.	.	2	0	1	5534.0	0.00	213E18	


BMI < 25 - GLOBAL CHI-SQUARE 1.93 ON 4 D.F. P = 0.75

Print Data Set WORK.TEMP1 Haplotype	Chi-Square	DF	P-value	Count	Control	Case	Odds Ratio Estimate	Lower 99% Confidence Limit for Odds Ratio	Upper 99% Confidence Limit for Odds Ratio	
h2314	0.20	2	0.9033	0	1339	1175	1.00	.	.	
	.	.	.	1	1577	1422	1.00	0.86	1.15	
	.	.	.	2	570	538	1.03	0.85	1.25	
h2132	0.75	2	0.6878	0	1954	1783	1.00	.	.	
	.	.	.	1	1253	1096	0.97	0.84	1.12	
	.	.	.	2	279	256	1.05	0.82	1.34	
h2112	4.05	2	0.1317	0	3444	3115	1.00	.	.	
	.	.	.	1	41	18	0.57	0.26	1.28	
	.	.	.	2	1	2	2.55	0.11	61.42	
h2312	1.14	2	0.5669	0	2985	2717	1.00	.	.	
	.	.	.	1	456	377	0.96	0.78	1.17	
	.	.	.	2	45	41	1.22	0.67	2.23	
h1132	0.16	2	0.9244	0	1936	1734	1.00	.	.	
	.	.	.	1	1292	1169	0.99	0.86	1.13	
	.	.	.	2	258	233	0.97	0.74	1.25	


25 < BMI < 30 - GLOBAL CHI-SQUARE 8.71 ON 4 D.F. P = 0.07


Print Data Set WORK.TEMP2 Haplotype	Chi-Square	DF	P-value	Count	Control	Case	Odds Ratio Estimate	Lower 99% Confidence Limit for Odds Ratio	Upper 99% Confidence Limit for Odds Ratio	
h2314	4.84	2	0.0887	0	1621	1560	1.00	.	.	
	.	.	.	1	1924	1772	0.94	0.82	1.07	
	.	.	.	2	704	594	0.86	0.72	1.03	
h2132	0.57	2	0.7507	0	2357	2195	1.00	.	.	
	.	.	.	1	1610	1464	0.97	0.86	1.10	
	.	.	.	2	282	267	1.03	0.81	1.31	
h2112	9.44	2	0.0089	0	4187	3873	1.00	.	.	
	.	.	.	1	56	53	0.98	0.56	1.71	
	.	.	.	2	6	0	0.00	0.00	296E95	
h2312	2.31	2	0.3146	0	3619	3317	1.00	.	.	
	.	.	.	1	572	563	1.09	0.91	1.30	
	.	.	.	2	58	46	0.85	0.49	1.48	
h1132	6.15	2	0.0462	0	2361	2091	1.00	.	.	
	.	.	.	1	1588	1522	1.09	0.96	1.23	
	.	.	.	2	299	313	1.21	0.96	1.52	


BMI > 30 - GLOBAL CHI-SQUARE 3.34 ON 4 D.F. P = 0.50


Print Data Set WORK.TEMP3 Haplotype	Chi-Square	DF	P-value	Count	Control	Case	Odds Ratio Estimate	Lower 99% Confidence Limit for Odds Ratio	Upper 99% Confidence Limit for Odds Ratio	
h2314	0.62	2	0.7324	0	550	405	1.00	.	.	
	.	.	.	1	632	448	0.94	0.74	1.19	
	.	.	.	2	212	145	0.92	0.66	1.29	
h2132	0.27	2	0.8739	0	792	565	1.00	.	.	
	.	.	.	1	506	370	1.00	0.79	1.26	
	.	.	.	2	96	63	0.91	0.58	1.44	
h2112	1.65	2	0.4382	0	1364	976	1.00	.	.	
	.	.	.	1	30	22	1.19	0.52	2.70	
	.	.	.	2	0	0	.	.	.	
h2312	1.59	2	0.4515	0	1141	833	1.00	.	.	
	.	.	.	1	224	146	0.85	0.61	1.18	
	.	.	.	2	29	20	0.97	0.42	2.24	
h1132	3.92	2	0.1406	0	773	521	1.00	.	.	
	.	.	.	1	529	410	1.20	0.95	1.51	
	.	.	.	2	93	67	1.11	0.70	1.75	


GLOBAL INTERACTION TEST - AGE	

Print Data Set WORK.TESTALLAGE1 ChiSq	df	Pr > Chi-Square	
5.15547	5	0.3972	

GLOBAL INTERACTION TEST - FAMILY HISTORY

Print Data Set WORK.TESTALLFHX1 ChiSq	df	Pr > Chi-Square	
2.21396	5	0.8188	

GLOBAL INTERACTION TEST - BODY MASS INDEX

Print Data Set WORK.TESTALLBMI1 ChiSq	df	Pr > Chi-Square	
12.6363	10	0.2447
