# Supplementary material for: Genetic Variation in the HSD17B1 Gene and Risk of Prostate Cancer
Source: PLoS Genet. 2005 Nov 25;1(5):e68. doi: 10.1371/journal.pgen.0010068 (PMC1287955; doi:10.1371/journal.pgen.0010068)
Supplement: Supplementary file 6 [file pgen.0010068.st006.doc]

AGE LESS THAN 65
	

Print Data Set WORK.TEMP1 SNP	Chi-Square	DF	P-value	Genotype	Control	Case	Odds Ratio Estimate	Lower 99% Confidence Limit for Odds Ratio	Upper 99% Confidence Limit for Odds Ratio	
rs676387(A)	0.24	2	0.8882	0	1528	1183	1.00	.	.	
	.	.	.	1	1068	819	0.98	0.83	1.14	
	.	.	.	2	210	155	0.96	0.71	1.29	
rs605059(G)	0.12	2	0.9434	0	756	584	1.00	.	.	
	.	.	.	1	1366	1057	1.00	0.84	1.20	
	.	.	.	2	634	493	1.02	0.83	1.27	
rs598126(A)	0.16	2	0.9238	0	735	568	1.00	.	.	
	.	.	.	1	1389	1057	0.99	0.83	1.18	
	.	.	.	2	669	507	1.02	0.82	1.26	
rs2010750(T)	0.50	2	0.7781	0	1070	771	1.00	.	.	
	.	.	.	1	1300	1025	1.05	0.89	1.24	
	.	.	.	2	447	348	1.03	0.82	1.29	

AGE GREATER THAN 65	

Print Data Set WORK.TEMP2 SNP	Chi-Square	DF	P-value	Genotype	Control	Case	Odds Ratio Estimate	Lower 99% Confidence Limit for Odds Ratio	Upper 99% Confidence Limit for Odds Ratio	
rs676387(A)	6.57	2	0.0374	0	3585	3199	1.00	.	.	
	.	.	.	1	2376	2299	1.08	0.98	1.19	
	.	.	.	2	456	479	1.16	0.96	1.39	
rs605059(G)	3.14	2	0.2082	0	1758	1708	1.00	.	.	
	.	.	.	1	3038	2860	0.96	0.86	1.07	
	.	.	.	2	1506	1346	0.91	0.80	1.04	
rs598126(A)	3.98	2	0.1370	0	1724	1681	1.00	.	.	
	.	.	.	1	3067	2892	0.96	0.85	1.07	
	.	.	.	2	1558	1384	0.90	0.79	1.03	
rs2010750(T)	5.62	2	0.0601	0	2304	2254	1.00	.	.	
	.	.	.	1	2935	2704	0.94	0.84	1.04	
	.	.	.	2	1104	961	0.88	0.77	1.02	


FAMILY HISTORY NEGATIVE

Print Data Set WORK.TEMP1 SNP	Chi-Square	DF	P-value	Genotype	Control	Case	Odds Ratio Estimate	Lower 99% Confidence Limit for Odds Ratio	Upper 99% Confidence Limit for Odds Ratio	
rs676387(A)	3.84	2	0.1469	0	3233	2817	1.00	.	.	
	.	.	.	1	2235	1983	1.00	0.90	1.12	
	.	.	.	2	407	419	1.16	0.95	1.41	
rs605059(G)	1.22	2	0.5442	0	1590	1459	1.00	.	.	
	.	.	.	1	2828	2497	0.95	0.84	1.07	
	.	.	.	2	1351	1211	0.97	0.85	1.12	
rs598126(A)	2.22	2	0.3290	0	1547	1438	1.00	.	.	
	.	.	.	1	2891	2532	0.93	0.83	1.05	
	.	.	.	2	1422	1258	0.96	0.83	1.10	
rs2010750(T)	1.86	2	0.3938	0	2205	1984	1.00	.	.	
	.	.	.	1	2706	2364	0.94	0.85	1.06	
	.	.	.	2	947	844	0.95	0.82	1.10	

FAMILY HISTORY POSITIVE	


Print Data Set WORK.TEMP2 SNP	Chi-Square	DF	P-value	Genotype	Control	Case	Odds Ratio Estimate	Lower 99% Confidence Limit for Odds Ratio	Upper 99% Confidence Limit for Odds Ratio	
rs676387(A)	1.21	2	0.5468	0	303	447	1.00	.	.	
	.	.	.	1	202	327	1.11	0.81	1.52	
	.	.	.	2	38	50	0.89	0.48	1.66	
rs605059(G)	0.21	2	0.8986	0	156	229	1.00	.	.	
	.	.	.	1	266	415	1.06	0.75	1.51	
	.	.	.	2	119	179	1.05	0.68	1.60	
rs598126(A)	0.13	2	0.9354	0	154	225	1.00	.	.	
	.	.	.	1	270	412	1.05	0.74	1.50	
	.	.	.	2	124	184	1.04	0.68	1.59	
rs2010750(T)	1.26	2	0.5336	0	212	293	1.00	.	.	
	.	.	.	1	251	404	1.09	0.78	1.51	
	.	.	.	2	83	115	0.90	0.57	1.43	


BMI LESS THAN 25	


Print Data Set WORK.TEMP1 SNP	Chi-Square	DF	P-value	Genotype	Control	Case	Odds Ratio Estimate	Lower 99% Confidence Limit for Odds Ratio	Upper 99% Confidence Limit for Odds Ratio	
rs676387(A)	0.23	2	0.8916	0	1894	1700	1.00	.	.	
	.	.	.	1	1276	1146	0.97	0.85	1.12	
	.	.	.	2	257	235	0.98	0.76	1.27	
rs605059(G)	0.60	2	0.7426	0	935	852	1.00	.	.	
	.	.	.	1	1640	1472	0.98	0.84	1.14	
	.	.	.	2	787	731	1.03	0.85	1.23	
rs598126(A)	0.21	2	0.9018	0	921	832	1.00	.	.	
	.	.	.	1	1675	1491	0.98	0.84	1.14	
	.	.	.	2	822	737	1.00	0.84	1.21	
rs2010750(T)	0.01	2	0.9931	0	1218	1090	1.00	.	.	
	.	.	.	1	1579	1428	1.00	0.86	1.15	
	.	.	.	2	587	543	1.00	0.83	1.22	

BMI BETWEEN 25 AND 30	


Print Data Set WORK.TEMP2 SNP	Chi-Square	DF	P-value	Genotype	Control	Case	Odds Ratio Estimate	Lower 99% Confidence Limit for Odds Ratio	Upper 99% Confidence Limit for Odds Ratio	
rs676387(A)	5.91	2	0.0522	0	2328	2055	1.00	.	.	
	.	.	.	1	1566	1499	1.09	0.96	1.23	
	.	.	.	2	303	315	1.20	0.95	1.50	
rs605059(G)	2.15	2	0.3421	0	1155	1102	1.00	.	.	
	.	.	.	1	1991	1867	0.98	0.85	1.12	
	.	.	.	2	972	853	0.91	0.77	1.08	
rs598126(A)	3.10	2	0.2126	0	1121	1088	1.00	.	.	
	.	.	.	1	2016	1882	0.95	0.82	1.09	
	.	.	.	2	1010	884	0.89	0.76	1.05	
rs2010750(T)	5.79	2	0.0554	0	1543	1473	1.00	.	.	
	.	.	.	1	1922	1762	0.95	0.83	1.08	
	.	.	.	2	717	587	0.85	0.71	1.01	


BMI GREATER THAN 30	


Print Data Set WORK.TEMP3 SNP	Chi-Square	DF	P-value	Genotype	Control	Case	Odds Ratio Estimate	Lower 99% Confidence Limit for Odds Ratio	Upper 99% Confidence Limit for Odds Ratio	
rs676387(A)	3.53	2	0.1709	0	762	511	1.00	.	.	
	.	.	.	1	526	404	1.18	0.94	1.49	
	.	.	.	2	92	67	1.13	0.72	1.77	
rs605059(G)	2.56	2	0.2775	0	373	277	1.00	.	.	
	.	.	.	1	659	488	0.96	0.74	1.25	
	.	.	.	2	334	209	0.83	0.61	1.14	
rs598126(A)	1.66	2	0.4371	0	364	269	1.00	.	.	
	.	.	.	1	653	482	0.99	0.76	1.28	
	.	.	.	2	347	223	0.87	0.64	1.19	
rs2010750(T)	0.32	2	0.8541	0	541	389	1.00	.	.	
	.	.	.	1	625	444	0.96	0.75	1.22	
	.	.	.	2	211	144	0.94	0.67	1.31	


INTERACTION TESTS- AGE
	
Print Data Set WORK.ALLAGETEST SNP	DF	Wald Chi-square	Pr > Chi-Square	
rs676387(A)	1	3.1110	0.0778	
rs605059(G)	1	1.3462	0.2459	
rs598126(A)	1	1.4863	0.2228	
rs2010750(T)	1	2.7375	0.0980	

INTERACTION TESTS- FAMILY HISTORY
	
Print Data Set WORK.ALLAGETEST SNP	DF	Wald Chi-square	Pr > Chi-Square	
rs676387(A)	1	0.0005	0.9827	
rs605059(G)	1	0.0212	0.8843	
rs598126(A)	1	0.0397	0.8422	
rs2010750(T)	1	0.2715	0.6024	

INTERACTION TESTS - BMI	

Print Data Set WORK.ALLBMITEST SNP	DF	Wald Chi-square	Pr > Chi-Square	
rs676387(A)	2	2.7865	0.2483	
rs605059(G)	2	1.9349	0.3800	
rs598126(A)	2	1.1832	0.5534	
rs2010750(T)	2	4.0512	0.1319
